# Similar rates of protein adaptation in *Drosophila miranda *and *D. melanogaster*, two species with different current effective population sizes

**DOI:** 10.1186/1471-2148-8-334

**Published:** 2008-12-18

**Authors:** Doris Bachtrog

**Affiliations:** 1 Department of Integrative Biology, University of California Berkeley, 3060 Valley Life Sciences Building, Berkeley, CA 94720, USA

## Abstract

**Background:**

Adaptive protein evolution is common in several Drosophila species investigated. Some studies point to very weak selection operating on amino-acid mutations, with average selection intensities on the order of *N*_e_*s *~ 5 in *D. melanogaster *and *D. simulans*. Species with lower effective population sizes should undergo less adaptation since they generate fewer mutations and selection is ineffective on a greater proportion of beneficial mutations.

**Results:**

Here I study patterns of polymorphism and divergence at 91 X-linked loci in *D. miranda*, a species with a roughly 5-fold smaller effective population size than *D. melanogaster*. Surprisingly, I find a similar fraction of amino-acid mutations being driven to fixation by positive selection in *D. miranda *and *D. melanogaster*. Genes with higher rates of amino-acid evolution show lower levels of neutral diversity, a pattern predicted by recurrent adaptive protein evolution. I fit a hitchhiking model to patterns of polymorphism in *D. miranda *and *D. melanogaster *and estimate an order of magnitude higher selection coefficients for beneficial mutations in *D. miranda*.

**Conclusion:**

This analysis suggests that effective population size may not be a major determinant in rates of protein adaptation. Instead, adaptation may not be mutation-limited, or the distribution of fitness effects for beneficial mutations might differ vastly between different species or populations. Alternative explanation such as biases in estimating the fraction of beneficial mutations or slightly deleterious mutation models are also discussed.

## Background

Researchers have made considerable progress in recent years to quantify rates of adaptive evolution in the genome using population variability data [1-6]. Many studies aimed at detecting adaptive evolution have applied the McDonald-Kreitman (MK) test [7] or modifications of it, which contrasts the number of polymorphisms within a species to the number of substitutions between species at two classes of sites, a putatively neutral and a putatively selected class. In protein-coding sequences these classes are usually synonymous and replacement sites [7].

Several members in the *Drosophila melanogaster *species group show high rates of adaptive amino-acid evolution. Using the MK test and its extensions, about half (and up to 95%) of all amino-acid mutations fixed between species are inferred to be driven by positive selection [1-4]. Some uncertainty in estimates of *α*, the fraction of amino-acid substitutions driven to fixation by adaptive evolution in *D. melanogaster *and *D. simulans *exists among different studies, reflecting in part the choice of loci and their chromosomal location and the populations surveyed, as well as the specific methodology employed to infer adaptive evolution (i.e. see ref. [8] for a discussion). However, estimates of *α *at X-linked loci in African, presumably ancestral populations of *D. melanogaster *and *D. simulans *are very similar between the two species. In particular, roughly 60% of amino-acid substitutions at X-linked loci are inferred to be adaptive in both a Zimbabwe population of *D. melanogaster *and a Madagascar population of *D. simulans *[3,9]. Both populations have similar levels of X-linked synonymous diversity, *π*_s _(about 3% [3,9]), indicating that they have similar effective population sizes. Comparable estimates of pervasive positive selection have been obtained in *D. americana*, a member of the *virilis *species group [10] which also has similar levels of synonymous diversity (around 2% [10]) and thus probably a similar effective population size (*N*_e_).

While adaptive protein evolution appears to be common in Drosophila, we know much less about the strength of selection (*s*) acting on beneficial amino-acid changes. Different studies and approaches have yielded very different estimates of *s *for amino acid mutations. In Drosophila, there is a correlation between rates of recombination and levels of nucleotide diversity [11], but the causes of this correlation are controversial [12,13]. Assuming that this correlation is entirely driven by beneficial mutations, Eyre-Walker [14] estimates the strength of selection of fixed mutations in *D. melanogaster *to be 350 <*N*_e_*s *< 3500. Macpherson *et al*. [15] also concluded that selection is strong (*s *~ 1%; i.e. *N*_e_*s *~ 10^4^-10^5^), by fitting a genetic hitchhiking model to patterns of genome variability in *D. simulans*. Similarly, Li and Stephan [16] used a likelihood approach to estimate selection parameters from patterns of single nucleotide polymorphisms in *D. melanogaster*, and infer selection to be strong (*s *between 0.05–0.5%). In contrast, Andolfatto [17] inferred much weaker selection by fitting genome variability data in *D. melanogaster *to a recurrent sweep model, and estimates *N*_e_*s *~ 40. Finally, by fitting MK-type data from *D. simulans *to a weak selection model, Sawyer *et al*. [4] estimate that the average scaled selection intensity of fixed mutations is only *N*_e_*s *~ 5. Thus, there is little agreement among studies on the average strength of selection of fixed mutations in Drosophila. However, if selection would indeed be on the order of *N*_e_*s *~ 5 [4], even modest changes in the effective population size among Drosophila species would have dramatic impacts on rates of adaptation between different lineages. That is because mutations are effectively neutral if *N*_e_*s *< 1, i.e. their fate is mostly governed by genetic drift and not selection [18].

Although the data are limited, there does appear to be a possible correlation between the level of adaptive evolution and population size: hominids appear to have undergone very little adaptive evolution [5,19], compared with Drosophila, while bacteria and viruses seem to show even higher rates of adaptive divergence [20,21]. This is to be expected given that large populations generate more mutations and selection is effective on a greater proportion of mutations [18]. This might mean that species with small population sizes are much less able to adapt to their environment.

There are, however, some problems with the very broad-brush nature of these patterns. The most fundamental difficulty is that many different aspects of the biology of these very different species compared are confounded. A more direct approach to estimate the influence of *N*_e _on rates of adaptation is to compare closely related species that differ in their effective population size, such as different members from the genus Drosophila.

Here, I report and analyze data from 91 X-linked protein-coding genes from *D. miranda*, to estimate rates of adaptation at the protein level. Synonymous site diversity in this species is substantially lower than in members of the *D. melanogaster *species group, only about 0.4% [22-24], suggesting a 5-fold lower current effective population size for *D. miranda*. Thus, if the average selection intensity for beneficial mutations in *D. simulans *is indeed in the order of *N*_e_*s *~ 5, as suggested by some studies [4], and the distribution of *s *is similar between species, a large fraction of the beneficial mutations fixed in *D. simulans *would behave effectively neutral in *D. miranda*. Previous studies based on many fewer genes scattered over different chromosomes have indeed found little evidence for positive selection operating on amino-acid mutations in *D. miranda *[23,25]. Applying MK-tests to this much larger data set, I find evidence for high rates of adaptive protein evolution, similar to those reported in the *D. melanogaster *species group. In addition, I show that genes with high rates of protein evolution harbor lower levels of synonymous site diversity, a signature of hitchhiking effects associated with linked beneficial amino-acid substitutions. These findings are discussed in comparisons with inferences drawn from *D. melanogaster*, in light of the effect of population size on rates of adaptation.

## Results

### Levels of polymorphism in *D. miranda*

Here, I study diversity at 91 X-linked coding regions in *D. miranda *(~1.1 kb on average), in a sample of 14 individuals. Table 1 gives an overview of the polymorphism summaries across the regions investigated, and their level of divergence to *D. pseudoobscura*. For locus-specific estimates of polymorphism statistics and divergence, see Additional file 1. A total of 489 synonymous and 144 replacement polymorphisms were observed. Average pairwise diversity is 0.62% at synonymous site, and roughly 15-fold less at replacement sites (0.04%, see Table 1). Similarly, divergence is lower at replacement sites compared to synonymous sites (Table 1). There is a general skew in the allele frequency spectrum towards rare variants, as measured by Tajima's *D *[26], both at synonymous sites and at replacement sites (Table 1). However, replacement sites are skewed more strongly towards rare variants compared to synonymous sites (*D *= -0.80 vs. *D *= -0.44, Table 1). Reduced diversity, reduced divergence and more low-frequency variants are all expected if amino-acid sites are under stronger purifying selection than synonymous sites [27]. The skew of synonymous sites could reflect non-equilibrium demography, or positive selection at linked sites (see below).

**Table 1:** Summary statistics for 91 X-linked protein-coding genes in a sample of 14 alleles of *D. miranda*.

	*π *(%)	D_xy _pse (%)	D_xy _aff (%)	D_xy _ANC (%)	Taj D
Synonymous Sites					
(23142 bp)	0.621	4.210	25.410	1.670	-0.444
Replacement Sites					
(73584 bp)	0.044	0.569	1.110	0.209	-0.798

Note – Dxy is average pairwise divergence from the outgroup (pse: D. pseudoobscura; aff: D. affinis; ANC: reconstructed ancestor of D. miranda and D. pseudoobscura)

Here, I use synonymous sites as a neutral marker in MK tests, and to detect hitchhiking effects associated with adaptive protein evolution. In Drosophila, synonymous codons are not used randomly, but instead some codons are used preferentially over a different codon encoding for the same amino acid [28,29]. Thus, synonymous sites are not necessarily free of selective constraints. Nevertheless, selection for codon bias is strongly reduced in *D. miranda*, and synonymous sites are evolving probably close to neutral in this species [25,30]. Thus, synonymous polymorphisms are a suitable "almost neutral" marker for tracking adaptive events in *D. miranda*.

### Adaptive protein evolution in *D. miranda*

The MK test and its extensions were used to test for adaptive protein evolution in *D. miranda *(Table 2). The fraction of amino-acid substitutions that have been fixed by positive selection (*α*) is estimated using three slightly different approaches [1,2,31]. Segregating slightly deleterious amino-acid mutations will bias the estimate of *α *downwards, because slightly deleterious mutations tend to contribute relatively more to polymorphism than they do to divergence, when compared to neutral mutations [2,32,33]. Slightly deleterious mutations segregate at lower frequencies than neutral alleles, and consistent with the idea of segregating deleterious amino-acid mutations, replacement polymorphisms do segregate at lower frequencies than synonymous mutations in *D. miranda *(Table 1). I attempt to reduce the effect of slightly deleterious mutations in my analysis, by also removing polymorphisms at a frequency of 10% or lower, at both synonymous and replacement sites (Table 2). If all polymorphisms are considered, estimates of *α *range from 12%–41%, depending on the estimation method used (Table 3). If low-frequency variants are ignored, I estimate *α *to be between 44%–61% (and the 95% confidence intervals do not overlap zero, Table 3). Thus, roughly half of the protein divergence between *D. miranda *and *D. pseudoobscura *is driven by positive selection. These numbers are in close agreement to estimates of *α *obtained in *D. melanogaster *and *D. simulans *if similar approaches are used to estimate *α *[1-3,9,14,31]; but also see [4].

**Table 2:** Count of synonymous and replacement polymorphism in *D. miranda *and divergence to *D. pseudoobscura*.

	Divergence	Polymorphism	
		all	f > 0.1
	
Synonymous	796	489	292
Replacement	396	144	56

Note – Dxy is average pairwise divergence from the outgroup (pse: D. pseudoobscura; aff: D. affinis; ANC: reconstructed ancestor of D. miranda and D. pseudoobscura)

**Table 3:** Proportion of amino-acid substitutions driven by positive selection (*α*)

	*D. pseudoobscura*		*D. affinis*	ancestor
			
method	all sites	excluding singletons	excluding singletons	excluding singletons
	*α *(95% CI)	*α *(95% CI)	*α *(95% CI)	*α *(95% CI)
Fay et al.	0.41 (0.19, 0.56)	0.61 (0.45, 0.74)	0.39 (-0.08, 0.69)	0.59 (0.25, 0.78)
Smith & Eyre-Walker	0.12 (-0.22, 0.38)	0.48 (0.28, 0.66)	0.32 (-0.17, 0.63)	0.54 (0.20, 0.75)
Bierne & Eyre-Walker	0.17 (-0.11, 0.36)	0.44 (0.19, 0.61)	0.41 (0.06, 0.61)	0.61 (0.28, 0.80)

Note – *α *is calculated using either *D. pseudoobscura*, *D. affinis *or a reconstructed ancestral sequence as the outgroup.

Average synonymous divergence between *D. miranda *and *D. pseudoobscura *is low, roughly only 4% (Table 1). It has been suggested that low levels of divergence can lead to upwardly biased estimates of *α *for two reasons [8,31]. First, advantageous mutations spread through a population much more rapidly than neutral mutations. Thus, the divergence time may have been to short for a stochastic steady state for fixations to have been reached, resulting in an upward bias in the estimation of *α *[31]. The divergence time between *D. miranda *and *D. pseudoobscura *is about 7-fold larger than the population coalescence time, very similar to that observed for *D. melanogaster *and *D. simulans *[9,34]. Thus, if low divergence leads to a bias in the estimation of *α*, a similar bias may be expected in the two species comparisons. Secondly, since only a single *D. pseudoobscura *sequence is used for outgroup comparison, some of the inferred divergence includes undetected segregating polymorphism, which could also result in an upward bias in the estimation of *α *[8]. To avoid potential biases in inferring the fraction of beneficial amino-acid fixations due to low levels of divergence, I also estimated *α *using divergence to a more distantly related species. To this end, *D. affinis *was employed, a species which shows about 25% synonymous divergence to *D. miranda *at synonymous sites (see Table 1). Interestingly, using *D. affinis *as an outgroup species results in similar, although slightly lower, estimates of *α *(i.e. 30%–40%, see Table 3). If *α *is estimated along the *D. miranda *lineage, using a reconstructed *D. miranda *– *D. pseudoobscura *ancestral sequence, estimates of *α *are increased (Table 3). Thus, while *α *may be overestimated for short divergence times, *D. pseudoobscura *appears sufficiently diverged from *D. miranda *to result in little bias in estimating *α *due to low levels of sequence divergence.

### Hitchhiking effects at fast evolving genes

Positively selected amino-acid mutations should leave characteristic signatures at surrounding genomic regions. One such signature is reduced neutral diversity surrounding sites that are targets of positive selection; i.e. selective sweeps [35-38]. Indeed, several recent studies have found reduced levels of synonymous diversity at genes with high rates of protein evolution in *D. melanogaster *[17,39] and *D. simulans *[15].

**Figure 1:**
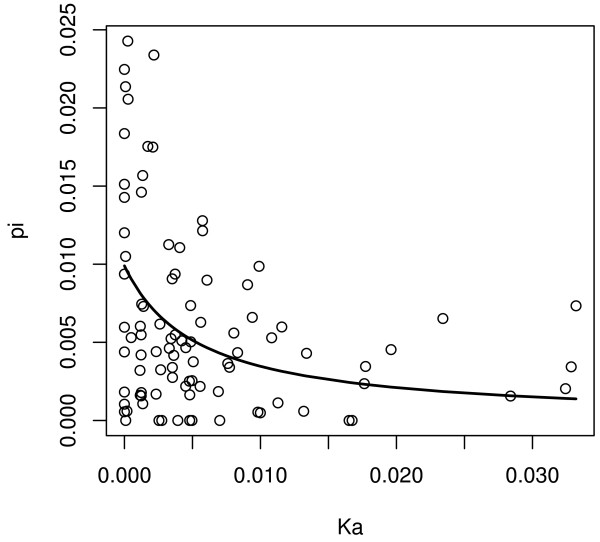
**Neutral diversity is reduced in fast evolving protein-coding genes**. The level of synonymous diversity (*π*_s_) is plotted against the rate of amino-acid evolution (K_a_) at X-linked loci in *D. miranda*. The solid line represents the least squares fit to a recurrent hitchhiking model.

Levels of synonymous diversity (*π*_s_) are reduced in protein-coding genes with high rates of amino-acid evolution (*K*_a_) in *D. miranda *(R = -0.265, p < = 0.01, rank correlation test; Figure 1), as expected if fast evolving proteins undergo more frequent adaptive evolution. I find no correlation between rates of substitutions at synonymous sites (*K*_s_) and *K*_a _(R = 0.0809, p < = 0.44, rank correlation test), suggesting little mutation rate variation among the regions studied.

**Figure 2:**
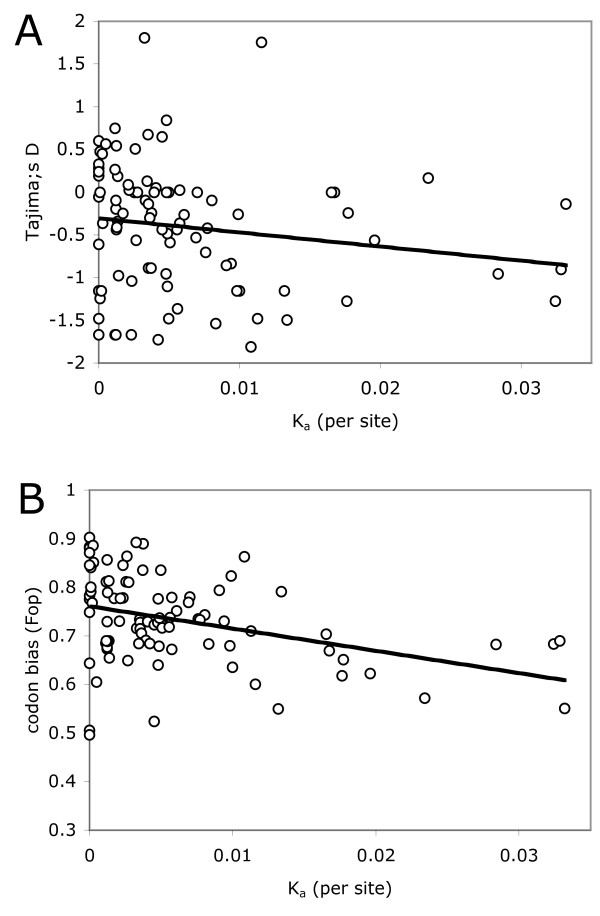
**An excess of rare polymorphisms and reduced codon bias in fast evolving protein-coding genes**. A. Tajima's D, a measure of the frequency distributions of mutations, is plotted against the rate of amino-acid evolution (*K*_a_) at X-linked loci in *D. miranda*. B. The frequency of optimal codons (Fop) is plotted against the rate of amino-acid evolution (*K*_a_).

The recurrent hitchhiking model predicts a stronger skew towards low-frequency variants at faster evolving genes [38,40]. In accordance with this expectation, I find a significant negative correlation between *K*_a _and Tajima's *D *(R = -0.2077, p < = 0.05, rank correlation test, Figure 2A). Recurrent hitchhiking is also predicted to reduce the efficacy of natural selection against very weakly selected sites, such as synonymous sites experiencing codon bias selection [41,42]. As a measure for codon bias selection experienced by each gene, I calculate the frequency of optimal codons (*Fop*), as identified in *D. pseudoobscura *[30]. Figure 2B shows that *Fop *is negatively correlated with rates of protein evolution (R = -0.3523, p < = 0.0008, rank correlation test), i.e. genes undergoing more adaptive evolution generally show less biased codon usage. This is consistent with the notion that adaptive protein evolution interferes with selection for codon usage at linked sites (but see also [43-45]).

### Estimating the strength of selection (s) under a recurrent hitchhiking model

Reduced diversity at synonymous sites, a skew toward low-frequency variants, and reduced selection for codon usage bias in fast evolving proteins are all features expected under recurrent adaptive amino-acid evolution. I fit a recurrent hitchhiking model to the relationship between synonymous site diversity (*π*_s_) and amino acid divergence (*K*_a_), to quantify selection parameters for beneficial mutations [13]. The diversity reduction under the hitchhiking model employed [13] depends on the rate at which beneficial mutations occur (*λ*), and their average selection coefficients (*s*). Assuming adaptive protein evolution solely accounts for the observed diversity reduction at fast evolving genes, and no heterogeneity in *α *among genes, as suggested in Drosophila [8,31], *λ *can be directly related to *α*, the fraction of adaptive protein divergence (*λ *= *αK*_a_*/2T*, where *T *is the species divergence time, see Methods). In Figure 1, I show the least squares fit of the *D. miranda *polymorphism data to this recurrent hitchhiking model. For comparison, I performed the same fitting procedure using a data set of 137 X-linked protein-coding genes from a sample of 12 *D. melanogaster *alleles sampled from a Zimbabwe population, which also shows a correlation between *K*_a _and *π*_s _[17]. Table 4 gives an overview of relevant population summary statistics for *D. miranda *and *D. melanogaster *and estimated selection parameters for beneficial amino-acid mutations. Note that the positive selection parameters inferred for *D. melanogaster *in this study differ slightly from those presented in ref. [17], where the same data set using a simulation-based method was analyzed. However, while the exact absolute values of selection parameters depend on the methodology used, it is the comparison between *D. miranda *and *D. melanogaster *that is the main focus of this paper. I estimate the neutral population mutation rate (i.e. in the absence of genetic hitchhiking effects) to be *θ *= 0.99% per site in *D. miranda *and *θ *= 2.81% in *D. melanogaster *(Table 4). The model also yields an estimate of the product *αs *= 1.2 × 10^-3 ^in *D. miranda *and *αs *= 1.2 × 10^-5 ^in *D. melanogaster*.

**Table 4:** Parameters inferred for positive amino-acid mutations in *D. miranda *and *D. melanogaster*

	*D. miranda*	*D. melanogaster*
K_a _(%)^a^	0.57	2.90
K_s _(%)^a^	4.21	14.91
*π*_s _(%)^a^	0.62	2.48
		
*ρ*/site/generation^b^	8.8E-08	3.3E-08
*μ*/site/generation^c^	5.8E-09	5.8E-09
*α*^d^	0.44	0.51
T = (K_s_-*π*_s_)/(2*μ*)	3,093,681	10,716,289
		
*θ*_0 _(%)^e^	0.99	2.81
2N_e_s* *α*^e^	1353.8	37.9
		
N_e _= *θ*_0_/(3*μ*)	568,851	1,613,218
2N_e_s	3103	74
s	2.7E-03	2.3E-05
*λ *= (α *K_a_)/(2T)	4.0E-10	7.0E-10
2N_e_s* *λ*	1.2E-06	5.1E-08
u_ben _= *λ*/(4*N_e_s)	6.5E-14	4.7E-12

^a ^estimated from polymorphism data

^b ^from True et al. (1996) and Oritz-Barrientos et al. (2006)

^c ^from Haag-Liautard et al. (2007)

^d ^estimated from polymorphism data using Bierne & Eyre-Walker (2004)

^e ^estimated from polymorphism data using Wiehe & Stephan (1993) model

In the recurrent hitchhiking model used, *s *and *α *(or *λ*) are conflated parameters and only their product can be estimated. However, I can infer *s *using an independent estimate of *α*. I estimate *α *= 44% for *D. miranda *and *α *= 51% for *D. melanogaster*, using a maximum likelihood method [31]. This value of *α *implies that *s *~ 2.7 × 10^-3^for *D. miranda *and *s *~ 2.3 × 10^-5 ^for *D. melanogaster*. Given estimates of *λ*, *N*_e _and *s*, we can also estimate the rate at which beneficial mutations arise per site and generation (*u*_ben _= *λ*/(4*N*_e _*s*), see Methods). The beneficial mutation rate is *u*_ben _~ 6.5 × 10^-14 ^in *D. miranda *and *u*_ben _~ 4.7 × 10^-12 ^in *D. melanogaster*. Thus, while the inferred rate of adaptation (i.e. the fixation rate of beneficial mutations) is similar between *D. miranda *and *D. melanogaster *(*λ *~ 4.0 × 10^-10 ^in *D. miranda *and *λ *~ 7.0 × 10^-10 ^in *D. melanogaster*, corresponding to *λ 2N*_e_*s *~ 1.2 × 10^-6 ^and *λ 2N*_e_*s *~ 5.1 × 10^-8^, Table 4), I estimate a lower beneficial mutation rate in *D. miranda *but larger average effects of beneficial amino-acid mutations.

## Discussion

### High rates of protein adaptation in *D. miranda*

If the rate of beneficial mutations and their selective effects are constant across species, larger populations are expected to show higher rates of adaptation, both because they generate more mutations and selection is effective on a greater proportion of mutations [18,46]. Kimura and Ohta [47] noticed that rates of protein evolution are rather similar across taxa with different population sizes, and argued against models of adaptive substitutions to explain protein evolution. Here, I compare rates of protein evolution in two Drosophila species that differ about 5-fold in their levels of neutral variability, but find little difference in rates of adaptive protein divergence between them.

Specifically, two independent types of evidence suggest that *D. melanogaster *and *D. miranda *both show high rates of adaptive protein evolution. First, application of the MK test suggest that a similar fraction of amino-acid mutations is driven to fixation in both species groups, and my estimate of *λ*, the rate of selective sweeps, is very similar between the two species. Second, both species show a correlation between rates of protein evolution and synonymous site diversity, as expected under recurrent adaptive protein evolution [17,39]. Fitting a recurrent hitchhiking model to polymorphism data suggests a higher value of *αs *in *D. miranda *than in *D. melanogaster*. Since *α *is estimated to be very similar between species, *s *is inferred to be larger in *D. miranda*.

What would we expect *α*, the rate of adaptive protein divergence, to be in *D. miranda *using selection parameters inferred from *D. melanogaster *but accounting for differences in their synonymous site diversity? The rate of adaptive divergence *K*_ben _can be calculated as *K*_ben _= 2N*u_ben _*2s*2*T*. Using estimates of *u*_ben _and *s *from *D. melanogaster *(Table 4), we would expect the rate of adaptive amino-acid divergence in *D. miranda *to be 0.107% per site. This means that only 18% of total amino acid divergence was driven by positive selection, i.e. *α *= 0.18. This value of *α *lies below the confidence limits we estimate for *α *in *D. miranda*, using MK approaches (Table 3). Thus, assuming that selection parameters are similar between the species, *D. miranda *displays higher rates of protein evolution than would be expected given its level of diversity. Below, I discuss several implications of this finding and possible explanations.

### Long-term vs. short-term differences in the effective population size

Levels of synonymous diversity are lower in *D. miranda *relative to *D. melanogaster *(see Table 4), suggesting a smaller effective population size in *D. miranda*. However, levels of diversity only contain information about the recent effective population size of a species, and it is possible that both species had similarly sized populations for much of their evolutionary history. Indeed, there is some evidence that *D. miranda *has experienced a reduction in its effective population size given reduced levels of diversity compared to its closest relatives, reduced selection to maintain codon bias, or mulitlocus patterns of diversity [22,23,30]. Thus, much of the adaptive evolution detected in *D. miranda *could reflect selection in a larger, ancestral population, or selection in *D. pseudoobscura *(which based on levels of synonymous polymorphism, is thought to have a larger population size [22]) since divergence also includes fixations along the *D. pseudoobscura *lineage. However, similar estimates of *α *are inferred, regardless of whether *D. pseudoobscura *or *D. affinis *is used as the outgroup species, or whether the reconstructed ancestral sequence of *D. miranda *and *D. pseudoobscura *is used (Table 3). While estimates of *α *might be biased upwards if divergence between species is low [8,31], a large fraction of amino-acid mutations along the *D. miranda *are clearly driven by adaptive evolution. Furthermore, if *D. miranda *has experienced a recent reduction in its effective population size, the estimate of *α *is likely to be biased downwards, due to segregating deleterious mutations (see below). Similar arguments of a reduced population size in *D. melanogaster *compared to its close relative to *D. simulans *have been invoked to account for lower levels of diversity at autosomal regions, decreased selection for codon bias and patterns of polymorphisms in *D. melanogaster *[48-50]. However, X-linked diversity appears similar between the two species [9,34], and estimates of adaptive protein evolution are comparable [8,9,31]. Thus, the exact dynamics of the long-term effective population size is unclear in both species, but both may have undergone a recent contraction in size.

While inferences of adaptive evolution based on the MK test are sensitive to assumptions about the long-term effective population size of a species, the correlation between synonymous diversity and rate of protein evolution provides an independent conformation of high rates of adaptive protein evolution in both species. This observation is less affected by fluctuations in the long-term effective population size, since reduced diversity in fast evolving proteins reflects the action of very recent selection (i.e. in the order of the population coalescence time). Interestingly, the rate of selective sweeps inferred from the observed correlation between *π*_s _and *K*_a _is very similar for the two species. Thus, despite uncertainties about the long-term effective population size in both *D. melanogaster *and *D. miranda*, several independent lines of evidence suggest similar and high rates of protein evolution in both species. It will be of great interest to study a diverse set of species that differ in their inferred effective population sizes, to better study the influence of population size on rates of adaptive evolution.

### Different distribution of fitness effects for beneficial mutations among species?

The expectation of less adaptation in smaller populations relies on the assumption that the mutation rate and the strength of selection for beneficial mutations are constant among species investigated. However, I infer that both the beneficial mutation rate and the strength of selection are different between *D. melanogaster *and *D. miranda*. But how plausible is such a scenario? In a species with a larger effective population size, a smaller fraction of mutations is effectively neutral, and one might expect such a species to be better adapted since more advantageous mutations will fix [18]. This could change the distribution of fitness effects because, as a species adapts, its fitness is expected to move closer to an optimum [51,52]. Thus, one could intuit that better adapted species have fitness distributions where beneficial mutations are weaker on average, but it may be harder to explain why the rate of adaptive mutations should be higher in that case.

There is some experimental evidence that the distribution of fitness effects is different between species that have different effective population sizes. Silander *et al*. [53] performed a mutation-accumulation experiment where bacteriophage populations were passaged through different population sizes. As expected, the small population size lines had lower fitness because they had accumulated more deleterious mutations. Surprisingly, about 15% of the mutations in the small population size lines were adaptive, in contrast to almost none in the large population size lines. The mean effect of mutations, however, did not seem to differ between lines. In comparisons between *D. miranda *and *D. melanogaster*, however, I infer that mutation rates are actually lower in the species with the lower population size, while fitness effects for beneficial mutations appear much larger.

### Bias in the estimation procedure to infer *α*?

**Figure 3:**
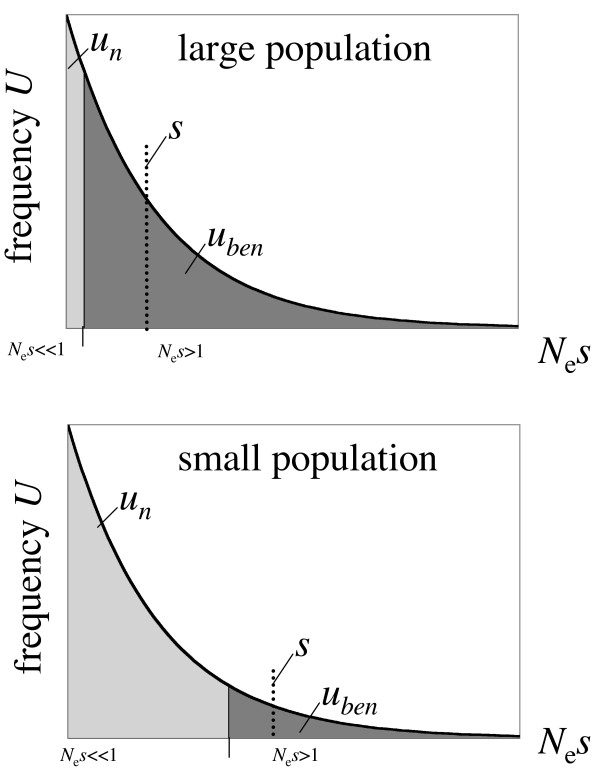
**The frequency of adaptive mutations (*U*) versus their strength, for large and small populations**. Assuming a fixed distribution of selective effects of mutations (*s*), large populations will have fewer effectively neutral mutations *u*_n _(light grey area) but more beneficial mutations *u*_ben _(dark grey area), than smaller populations. The mean effect of a beneficial that can become fixed in the species will be larger in a smaller population.

It is also possible that the distribution of beneficial amino-acid mutations is conserved across species, but that biases in estimating *α *result in a substantial underestimation of the rate of adaptive amino-acid divergence in *D. melanogaster*. A schematic model of this effect is shown in Figure 3. Assume the rate of mutation and the strength of selection for adaptive amino-acid changes are constant between species. Because of its larger population size, fewer advantageous mutations will be effectively neutral in *D. melanogaster *and many adaptive substitutions of weak effect can fix in this species. In contrast, many more mutations are effectively neutral in *D. miranda*, but the average effect of the mutations incorporated would be larger. This would cause us to infer a larger beneficial mutation rate, but smaller selection coefficients in *D. melanogaster*, as observed.

While this model could account for the observed differences in rates and effects of beneficial amino-acid mutations, it would also predict that *D. melanogaster *shows much higher rates of adaptive protein evolution than *D. miranda*. However, my estimate of *α *is very similar between the two species. This discrepancy could potentially be explained by a bias in estimating *α *from MK tables, which assumes that all amino-acid polymorphism segregating in the population are neutral [7]. However, if the fraction of beneficial amino-acid mutations is high and selection is weak, a substantial proportion of the amino-acid polymorphism segregating in the population might actually represent beneficial mutations. For example, Sawyer *et al*. [4] estimate that about half of all amino acids segregating in a population sample from *D. simulans *are beneficial. Including these mutations into MK tables will downward bias our estimate of *α*. Thus, if only very few amino-acid mutations are neutral in *D. melanogaster *(i.e. the majority is either deleterious or slightly beneficial), then a large fraction of amino-acid mutations that segregate in the population will be beneficial (once removing the low-frequency class), resulting in an underestimate of *α *using the MK framework employed here. In contrast, a larger fraction of amino-acid mutations will be effectively neutral in *D. miranda*, due to its smaller current population size, and thus a much smaller proportion of the observed amino acid polymorphisms are beneficial. This will cause the estimate of *α *to be much closer to its true value in *D. miranda*, while *α *might be substantially underestimated in *D. melanogaster*.

Consistent with a substantial fraction of amino-acid mutations segregating in *D. melanogaster *being beneficial, amino-acid polymorphism is less reduced relative to synonymous sites in *D. melanogaster *compared to *D. miranda *(*π*_a_/*π*_s _= 0.08 in *D. melanogaster *vs. *π*_a_/*π*_s _= 0.07 in *D. miranda*). Purifying selection against deleterious amino-acid mutations would be more effective in a larger species, predicting the opposite patterns, while the *π*_a_/*π*_s _ratio should be similar among species if amino-acid polymorphisms are neutral. In fact, if half the amino-acid polymorphisms are removed from *D. melanogaster *(i.e. they are assumed to be beneficial instead of neutral), the maximum likelihood estimate of *α *becomes closer to 0.8 in that species. In this case, we would conclude that *D. miranda *shows reduced rates of adaptation, in accordance with expectations based on its smaller current effective population size. While this model could qualitatively account for the observed patterns with regards to population size and adaptive evolution, it remains to be seen whether it can also quantitatively do so.

### The influence of deleterious mutations

In the above considerations I assume that both the excess divergence at amino acid mutations relative to polymorphisms as well as the correlation between neutral diversity and protein evolution are entirely driven by beneficial mutations. However, for both interpretations, weakly deleterious mutation models pose a potential problem.

Slightly deleterious nonsynonymous mutations can yield biased estimates of *α *using MK methods, and the direction of this bias depends on the demography of the population. As mentioned before, if the population size has been relatively stable, the estimate of *α *is likely an underestimate, because slightly deleterious amino-acid mutations tend to contribute relatively more to polymorphism than they do to divergence when compared with neutral mutations. However, slightly deleterious mutations can lead to an overestimate of *α *if population sizes have expanded, because mutations that might have been fixed in the past, when the population size was small, no longer segregate as polymorphisms. Even fairly modest increases in population size can create artifactual evidence of adaptive evolution [33]. Thus, it is possible that *D. miranda *has undergone a recent population size expansion (i.e. its current *N*_e _is larger than the ancestral one) and that *α *is therefore overestimated. However, as mentioned above, if anything *D. miranda *appears to have gone through a population size decrease based on several consistent patterns of polymorphisms and divergence [22,23,30]. This suggests that *α *has not been overestimated in *D. miranda *because of a population size increase.

I invoke recurrent hitchhiking to explain the observed correlation between *K*_a _and *π*_s _in *D. miranda*; recurrent beneficial amino acid mutations reduce variation more frequently at faster evolving genes, causing a reduction in codon bias and an excess of low frequency variants. It is, however, formally also possible to explain the observed correlation between *K*_a _and *π*_s _solely by deleterious mutations models. The removal of recurrent deleterious mutations from the population reduces the local effective population size, causing regions with higher deleterious mutation rates (or lower recombination rates) to harbour reduced levels of neutral diversity; i.e. background selection [54,55]. A reduction in *N*_e_, in turn, predicts an acceleration in the rate of accumulation of very weakly deleterious mutations [32,56], including possible unpreferred codons or slightly deleterious amino acid mutations. Weak background selection can also distort the allele-frequency spectrum of neutral mutations towards rare variants [56,57]. Thus, selection against weakly deleterious mutations can by itself account for reduced levels of neutral diversity associated with increased rates of accumulation of slightly deleterious amino-acid mutations [32,56], without invoking any beneficial mutations.

In *D. miranda*, the correlation between *K*_a _and *π*_s _is stronger than in *D. melanogaster*. This would suggest a larger fraction of slightly deleterious amino-acid mutations in the roam of weak background selection at some genes in *D. miranda*, causing a stronger reduction in *N*_e _and *π*_s _and higher rates of accumulation of deleterious amino-acid mutations. Again, a distribution of fitness effects against deleterious amino-acid mutations may in principle explain the pattern, if a larger fraction of deleterious amino-acid mutations in *D. melanogaster *is selected against efficiently. That is, only few amino-acid mutations with a relatively small range of negative selection coefficients cause weak background selection in *D. melanogaster*, while the majority of deleterious amino-acid mutations are selected against effectively. In contrast, more slightly deleterious amino-acid mutations segregate in *D. miranda*, resulting in a larger variance in the amount of weak background selection among genes. It is also possible that there is more heterogeneity in rates of recombination among the loci sampled in *D. miranda*, since physical and genetic map positions are not known for the loci investigated. Recombination rate variation may result in heterogeneous levels of background selection along a chromosome, and thus cause the local *N*_e _(and the efficacy of selection) to vary among regions. Indeed, there is evidence for recombination rate heterogeneity in *D. pseudoobscura*, where mild "recombination hotspots" were discovered [58]. Unfortunately, no genetic map exists in *D. miranda*, preventing us from directly investigating the effect of recombination rate heterogeneity on levels of polymorphisms in this species. However, as Andolfatto [17] points out, there are several empirical arguments against weak background selection causing the observed correlation between *K*_a _and *π*_s_, such as no general elevation of *K*_a _in regions of the Drosophila genome with reduced recombination rates [42,59], and no effect of gene density on rates of protein evolution [17].

### More complicated models of selection

In a series of papers, Gillespie examines the role of population size in population genetical models of molecular evolution, using extensive computer simulations [60,61]. Gillespie has defined three domains based on their rates of substitutions, which he terms the Ohta domain (the rate of substitution decreases with increasing population size), the Kimura domain (the rate of substitution remains close to the mutation rate) and the Darwin domain (the rate of substitution increases with increasing population size). Not surprisingly, he finds that nearly neutral deleterious mutation models all fall within the Ohta domain where the rate of evolution is inversely proportional to population size. He also finds that the normal-shift model (a model of positive selection) appropriately falls within the Darwin domain, but that the rate of substitution does not linearly increase with populations size and is substantially reduced relative to the expectation under the independence among sites model. Surprisingly, Gillespie also finds that the fluctuating selection, neutral, and overdominance model all lead to the Kimura domain, where the rate of molecular evolution is independent of the population size. In addition, adaptive evolution can actually cause the rate of substitution of deleterious alleles at a linked locus to increase with increasing population size [60,62]. Thus, the associations between *N*_e _and rates of evolution might be complex, and intuitions drawn from very simple models of positive and negative selection might not apply to natural populations. Future work will be needed to assess how more complicated selection models, including selection from standing variation or mutation selection balance [63,64], and interactions between beneficial and deleterious mutations will influence rates of molecular evolution and variation.

## Conclusion

Adaptive protein evolution is common in several Drosophila species investigated, but little is known about underlying selection coefficients of beneficial amino acid mutations. If average selection intensities are very weak, as suggested by some studies, even modest changes in the effective population size between species may have drastic impacts on rates of adaptation between lineages. Here, I estimate that a similar fraction (~50%) of amino-acid mutations is being driven to fixation by positive selection in two species of *Drosophila *that differ roughly 5-fold in their effective population sizes. Genes with higher rates of amino-acid evolution show lower levels of neutral diversity in both species, a pattern predicted by recurrent adaptive protein evolution. Thus, while adaptive amino-acid evolution is common in the genus *Drosophila*, modest changes in population size appear to have little influence on protein adaptation.

## Methods

### Survey of coding regions

A total of 91 X-linked coding regions were surveyed in this study with a sample size of 14 *D. miranda *alleles. The following strains of *D. miranda *were used in the analysis, with their population origin given in parenthesis: 0101.3, 0101.4, 0101.5, 0101.7 (Port Coquitlam, BC, Canada); 0101.9, MA28, MA32, MA03.1, MA03.2, MA03.3, MA03.4, MA03.5, MA03.6 (Mather, CA, USA); SP138, SP235, SP295 (Spray, OR, USA); MSH22, MSH38 (Mt. St. Helena, CA, USA).

The *D. pseudoobscura *genome sequence was used to provide estimates of divergence [65]. For a subset of 51 loci, the orthologous *D. affinis *allele was surveyed (strain 14012-0141.01 or 14012-0141.02). All genes were selected randomly with respect to gene function. Information about the specific loci surveyed and primers used can be found in Additional File 1.

Each ~1200 base pair region was PCR-amplified from genomic DNA extracted from single male flies, and primers and nucleotides were removed using Exonuclease I and Shrimp Alkaline Phosphatase. Cleaned products were sequenced on both strands with the original PCR primers and internal sequencing primers, using Big-Dye (Version 3, Applied Biosystems) and run on an ABI 3730 capillary sequencer. Sequence traces were edited using Sequencher (Gene Codes) software and multiple sequence alignments were generated using MUSCLE http://www.drive5.com/muscle/ with protein-alignment-assisted adjustments to preserve reading frames. Sequences have been deposited in Genbank under accession numbers (XXXX-XXXX).

For comparison with *D. melanogaster*, I used a published dataset of 12 individuals sequenced from a Zimbabwe, Africa population and their divergence to *D. simulans *[17].

### Polymorphism and divergence analysis

The estimated number of synonymous sites, nonsynonymous sites, average pairwise diversity (*π*), average pairwise divergence to *D. pseudoobscura *(*D*_*xy*_), as well as counts of the number of polymorphisms (*S*) and the summary of the frequency distribution of polymorphism frequencies, Tajima's *D *(Tajima 1989), were calculated using a library of Perl scripts ("Polymorphorama") written by the author and P. Andolfatto. The number of nonsynonymous and synonymous sites were estimated using the method of [66]. Average pairwise diversity (*π*) and divergence (*D*_*xy*_) estimates were corrected for multiple hits using a Jukes-Cantor correction [67]. Multiply hit sites were included in all analyses but insertion-deletion polymorphisms and polymorphic sites overlapping alignment gaps were excluded. For lineage-specific estimates of divergence, I reconstructed a *D. miranda *– *D. pseudoobscura *ancestor sequence (ANC) by maximum likelihood, using *D. affinis *as an outgroup, as implemented in the *codeml *program of PAML. Locus-specific estimates of levels of diversity and divergence can be found in Additional File 1. To estimate codon bias selection at each gene, the frequency of optimal codons (as inferred from *D. pseudoobscura*, see [30]) is used.

The fraction of amino acid mutations driven by positive selection, *α*, is estimated using three slightly different approaches based on the MK test [7], as implemented in the DoFEv2 software package (kindly provided by A. Eyre-Walker). The number of divergent sites were corrected for multiple hits using a Jukes-Cantor correction. I also exclude singleton mutations, to minimize the downward bias in inferring *α *due to segregating deleterious amino acid mutations.

### Estimating recurrent hitchhiking parameters

Following [17], I use the relationship between synonymous site diversity (*π*_s_) and amino acid divergence (*K*_a_) to quantify recurrent selection parameters. I use the analytical approximation of [13], to jointly estimate the strength of selection (*s*) and the rate of adaptive substitution per site per generation (*λ*). The expected nucleotide diversity at neutral sites is

(1) $$E(\pi )=\frac{\theta \rho }{\rho +k\gamma \lambda },$$

where *θ *is the population mutation rate, *ρ *is the recombination rate per site per generation, *k *is a constant ≅ 0.075, and γ (= *2N*_e_*s*) is the intensity of positive selection (where *N*_e _is the effective population size of the species and *s *is the strength of selection). Like [17], I assume that the rate of selective sweeps in the neighborhood of a focal neutral site is determined by the local rate of amino acid substitution at a gene *K*_a_, and that some constant fraction of amino acid divergence at each locus, *α*, was driven to fixation by positive selection. Therefore, the rate of selective sweeps due to recurrent adaptive amino acid substitutions at a locus is *λ *= *αK*_a_/2*T*, where *T *is the divergence time in generations between *D. miranda *and *D. pseudoobscura*. Equation (1) can be re-written as

(2) $$E(\pi )=\frac{\theta \rho }{\rho +k2N_{e}s(\alpha K_{a}/T)},$$

and equation 2 allows levels of neutral diversity to be directly related to the extent of adaptive amino acid divergence at each locus (i.e. *α K*_a_).

Average recombination rates in *D. pseudoobscura *are higher than those observed in *D. melanogaster*; average recombination rates across the X chromosome in *D. melanogaster *equal 3.3 cM/Mb [68], while the average recombination rate across the X chromosome in *D. pseudoobscura *is 8.3 cM/Mb [69]. Estimating $T=\left({\bar{K}}_{s}-\theta \right)/2\mu$ and *N*_e _= *θ*/3*μ *require an estimate of the neutral mutation rate, *μ*. I assume that $\hat{\mu }$ = 5.8 × 10^-9 ^per generation, the estimated average rate for single nucleotide mutations from *D. melanogaster *mutation-accumulation lines [70]. With these parameter estimated and *π*_s _and *K*_a _for 92 loci, a least-squares method is used to find the values of *θ *and *αs *that minimize the sum of the squared deviations between observed *π*_s _and *E(π) *predicted by the model, using the R statistical package.

## Authors' contributions

DB designed the study, analysed the data, and wrote the manuscript.

## Supplementary Material

Additional file 1 Supplementary Materials. This document contains tables with sequences of PCR primers and internal sequencing primers used in this study and additional results referenced in the main text. Click here for file
