# Supplementary material for: Similar rates of protein adaptation in Drosophila miranda and D. melanogaster, two species with different current effective population sizes
Source: BMC Evol Biol. 2008 Dec 18;8:334. doi: 10.1186/1471-2148-8-334 (PMC2633301; doi:10.1186/1471-2148-8-334)
Supplement: Additional file 1 — Supplementary Materials. This document contains tables with sequences of PCR primers and internal sequencing primers used in this study and additional results referenced in the main text. [file 1471-2148-8-334-S1.doc]

**Supplementary Materials.**

Supplementary Table 1. PCR primers and internal sequencing primers used in this study.

| locus |  | forward | reverse | seq_int_for | seq_int_rev |
| --- | --- | --- | --- | --- | --- |
| CG10990 | XL | TCCCATAACAAACAAGCCAAC | TCAGCTCATGATGATAGTGCG | TACGAGGACGAGAACGATCC | AATCGAGGTAATCCGTTCCC |
| CG11122 | XL | ACCTCAAGAAGCTGGAGAACC | TCATGTTGTGCTTGGACAGAG | AGCAGATGTACCAGAAGCGG | CCGTGAGGTAGTTGTCCTCC |
| CG11409 | XL | AACGTGAAGAGTCTGAATGGG | GACTTGAAGGTTTCGTTGCTG | ATCTCGAGGAGCTGCAGAAG | ATGGTGGTCAGCGAGAAGAC |
| CG11759 | XL | CGCCTGAACAAACAGCTAAAG | GATTAGATAAGCGGGCGATTC | ACAAGGTGAAGGGCATGTTC | TCCTGATACTTGAACGCACG |
| CG12132 | XL | ATAAGTGTGGCCTACGACGTG | TAGTAGCGCAGAAAGCTGAGG | CTTCCTAAACACCTGCTCCG | ACCACACGAACTCCTTGTCC |
| CG12149 | XL | GTGCCCAGCACACTCTTCTAC | ATTTCCTTGAGCTTCTCCTCG | CGAGACTCACCCAGAGTTCC | TAGTTGAGCATCCCCTCGTC |
| CG12199 | XL | TTCAAGGGCCTCAACATACTG | GAGGAGGCTCTTCTGCATTTC | ACTACGTGATCTCGAAGCGG | TGTACACCCAGGTGACGTTC |
| CG12467 | XL | ACGAACTAGGAACCCGAGATG | TGCTCAGTGTGTGCGAGTG | GCTGAAGGAGGTTCAAGAGC | TCTAACACGTTCAGCGCATC |
| CG12737 | XL | CTGATGATGAAAACGCAGATG | CTGTTCACCATATCCTCGGTG | TGAAGCTGTCGCAGAAGATG | TCATGATGCGGTAGCAGAAC |
| CG13366 | XL | CAGCGAGGTATCAGTAGCCTG | CTCCTTTTGCGTCTTGTTGTC | CCAAGGAGGATATTTCGCAG | AAGTCCGCTTCCAGTTCCTC |
| CG1372 | XL | GAACCAGACACTGAGCAAACC | GCGTGATTCTCGTAGATGCTC | AGTGAGGAATCTCAGCGAGC | GATAGACGACACCCTGCTCC |
| CG1517 | XL | ATACCGGACACCTTTTGTTCC | CAGCTTCTTGATGTCCTCGTC | GGATGCTGGTGATGACTGTG | CGAATGCTTGTAGTAGCCCC |
| CG1554 | XL | TATTCCACATTAACAAGCGCC | CTGGAAGATCTCGCAAATGTC | CTCGAAGAACGTGACACTGG | CTGATCCTCCGATATCACCG |
| CG1634 | XL | CCCCTATTTCACCGTAGAACC | GGTAGTGCAGGACTCGCTATG | ATCCTGGACGTGACCTTCTC | ATCTCCAGATTTCCGTCGTG |
| CG17758 | XL | ATTGGCAGCATGATCTATTCG | CGTAGAAGTTCAGCTGGATGG | CATCCTGTACGTCATGTGGC | ATGAGGATGCCCACAAAGAG |
| CG2025 | XL | ATCTAGCAGCTCCTCCGATTC | CTTGCTGTTGAAGACCAGCTC | CAAGGACAACGTGGACGAC | TCACGAAGAACACTTGCTCG |
| CG2175 | XL | CGGATAGCTTTAACCTCGGAC | CATTTTTGCTTCGTTTTGCTC | TATCCGAGGTGCGAGTTAGG | ATCTTCAGCTCCGCAATGAC |
| CG2194 | XL | GAACTTCCTGCCTTTGGTTTC | TTCGACCATATCGGGTATCTG | GGTGGATAGGACCACAATGC | GATTCTCAAACTTCAGGCCG |
| CG2841 | XL | CTTCAAGCTCTGCCAGACATC | ACTGGTGGTGACTGTTCCTTG | CAGTCGCTCTGACTCCTTCC | CTCGGTCAGGCTATTCTTGC |
| CG2984 | XL | AACGAGCAGGATGTGATGAAC | TCCAGCTGTGTGAGGGAGTAG | ACAGGTGGTAGTCGAGTCGG | AGAATGACCACATTTTCGCC |
| CG3003 | XL | GAAAGCTTCAATCTGTGGGTG | GATGAAGGAGGAGGACTAGCG | TCCACAGCATAACTCCCCTC | AAGAGCTAACAGACACGCCG |
| CG3078 | XL | GCCGTGAGTCGATCAATAGC | ATGTATGCCTGCTCCGTACC | GAGGCTTAGTTGCTCCCAGG | GAGCAACTCCAGTCGCTACC |
| CG3093 | XL | CTGATCAGCCTGGACTTTGAG | GAGCAATTTTGGCTCGTACTG | ACAAGCGGCCAATTATCAAC | AAAGATCGATGAGCCACACC |
| CG3193 | XL | CCCGCATCATCTACAAGTACG | CTCTACGCCATCGTCAGAGAC | ACTTGATGCCACACAAGCAG | AGGTTACGCAGTTTTCGGG |
| CG32527 | XL | AGTGGTATAAATTGCCGGCTC | AGGACGAGGAGGAAAGTGAAG | CTCTAGCGAGGAGGATGTGG | CTACCTCCAGGCTGTCCATC |
| CG32560 | XL | GTGTACGAGGCGAAGAATCTG | CTGTTCGAGGAAATCGTTCAG | ATTATAAGCGCGTCTGCGAG | TTAGCTTCAGGAATGCCTCC |
| CG32562 | XL | TGACCTGCACTGTGAGCTATG | CAGATAGCTTGTTTCCGGATG | ACATGTGTCCGGAGAAGGAG | TGCGTGAATATTGCTTGAGG |
| CG32704 | XL | GCAATGGAGCAACTTTCTGAC | TTGATCGGATAATCCCAGATG | CCTGAAGATGTACTCGGAACG | ACCAGATCATAAAGGCCGTG |
| CG3620 | XL | CAGAGCTCCATTGATTTCGTC | AGTTGTTTGATTTGAGCAGCC | GAATCCGGTCTACAACGAGG | TTCACAAACAGCATGGGAAG |
| CG3806 | XL | GACTGTACACATGATTGGGGG | AGGTCACCCATTTTGGGTATG | TGCTCATCAACGAGGAGTTG | TCAGGAACGAGTGGGTAAGC |
| CG4557 | XL | ATTCCACAAAGACCATGAAGG | TGGACATACTCCTGGTTCTCG | TGGTGAATCGCATCAGTGAG | CTGGAACTTCTTCTCCAGCG |
| CG4857 | XL | GTAGCAATGGAAATGGCAATG | TTGGTGTACGCCGTGTAGTAG | GCAGCTGGTCAAGTACATCG | GAGGCAGAGTTCTGTTTGCC |
| CG6775 | XL | AGAGGTGACCACCACCAGAG | CTCCACTGTCTCCCTTGTCTG | GCTGATCGAAAAGGTGATCG | TTGGGGTTTCACTTCCTCAG |
| CG7092 | XL | TGGCTGATCTTCTGTTCAAGC | AGTTGTACTCGTTGACGTGGG | CATTCTGTTGGGCTTCTTGG | TCGTGGTAGAACAGTCGCAG |
| CG8062 | XL | TTCTTTCCGTATGTGTCCACC | ACACCGATCATCACCTCGTAG | GAGATCACAGAACCGGGAAC | TCCCGATTGAAGTAGTTCGG |
| CG8128 | XL | GGTCAGAACCTGCTCTGTCTG | ATGTCTGCCGTTTCGTTGTAG | ACGACCAGCTTCTTCGAGAC | GAAGCGTTTCATGGCATAGG |
| CG8465 | XL | TGCCTGCAATAATTTGAGTCC | AAAGACTCTCGGTCTTGAGGG | CCTAGAGATGGTGGGCAGAC | TGCCTTTGATGCTGATTCTG |
| CG8590 | XL | GAGAGATCAGTCAGCTGGTGG | ATACGCTGCTCGAGAATGTTG | CATCACACAGGCCAATATGC | AGCTGGGTGAGCTCCTTCTC |
| CG9059 | XL | ATGACATCAAATATCCGGCAG | GACGAATCTCCACGCTGAAC | TGCAGATGCAGCTACAGGAG | AGCCGACAATTTCTGTGACC |
| CG9198 | XL | AAGCAATTGTTTGCTTTGTGC | TCGTAGTCCACATCTCCATCC | ATTGAGCGTTCATCTGGAGG | ACACAGTTCTCCATCTCGGG |
| CG9774 | XL | CATGGTGATGGATTTTATGCC | GACCTTCTCGCTGATGTTGAG | CAAGAACTCGCTCAGCTTCC | TTTTCGTCCCTCTCGATGTC |
| CG9900 | XL | CGAAATCGACAGTTTCCAAAG | TAGGTCCCGTACTGTTCGATG | ATGTACAGGCCCATCTCCTG | GACAGGCTTGAGGATAAGCG |
| CG10107 | XR | GGAGTTTAAGAAGCGCCAGAC | GATGGAAAGGTCACACCACTC | ACTGATCACCTTCACCCTGC | TCAGTTCACTGGCAGATTCG |
| CG10129 | XR | TACAACATGGCCAAGGAAATC | TCAGTGGATTGATCTTCGGTC | ACAAGCCCAAGGAGATTGTG | TCGACTACTTCCTCGCCATC |
| CG10236 | XR | GAAAACACTGACCACTCGACC | CCCTTGAAGGTCTTCTTCGTC | CTGTGTTCTCCGTCGAGATG | TCGAACTTGATGTCCGTCAG |
| CG11250 | XR | CAGCACGTACAGCAGTCTGAG | AAATGAGTCTTCTTGGTGGGC | GCTTCTATCGCTGGATGACC | AGTTGGCTATGCTCTGGTCG |
| CG12734 | XR | GATGCAGGAGAACGTTCAGAG | GGAGTCGATGTCTTTGAGCAG | ACTGGACACCCAGACCAAAG | AGTTTCTCCAGATTGTGCCG |
| CG12982 | XR | TTACTCACTATGCCGGAGGTG | AAATGCTCGTAGCGTTTGAAG | GAAATTACGGAGCATGGAGC | TCAAATATGGGAAAGAGCCG |
| CG12983 | XR | TCCAGAAGTTCAACGTGGTTC | CGAAGATACCCAAACGATCTG | GCGGCAGAGTAGACTGGAAC | CCTCGAAATCCTGTACCCTG |
| CG13900 | XR | CTACACGGAGGACACGAAGAG | CAATTGAGAGGTACTGCTCGC | TGCTGCGCATCTATGATCTC | CGTAGTCCAGCAGTGTGGTG |
| CG13908 | XR | ATCATAGTCGCCACACCTCAC | CTCGGTTGGAAGGGATAGTTC | AACAATTCCATCAAGTCGGC | TCTAGAGTAGAGCCTGCCGC |
| CG13917 | XR | ATACCATGGAGTCCCTGCAC | CATGTAGGCAATGATCTGCTG | ACATCCAGACCGATCTCAGC | TGCAGGAACATCTTGAGCAG |
| CG14060 | XR | ATGATGATCTAAAGCCCGACC | CCCTTGATGGTGTCGTAGATG | AATCAGGCGGTAAACGTGAC | TCGTAGATGGATTCCGGTTC |
| CG14967 | XR | TACATCGCTGAAGAAACCACC | GACAGCTGCGTCTCCTTGTAG | TTCAGGACAAGGACGAGACC | CCTCTGTTATGCTGTTGGGG |
| CG15804 | XR | CTTCAAGTACGTAAGCGCCAC | TCTCGTTGATGACCTTGATCC | AACCTCAGGTGGAAACCCTC | AGATGTTCAGAGCCTCACGC |
| CG17149 | XR | ATCAACGACGATGACTCCAAC | TGCATCTCTTGCATGCTTATG | ACCGCGTGTTCCTTAACATC | CACCGATGACAATCACCTTG |
| CG17150 | XR | GCTCTGACGCTGAAAAATGTG | GATGATGTTCACGAGATTGGC | ATGTGCTCGTGAAGAACGTG | ACCTGGGTGAAGACCTTGTG |
| CG17286 | XR | CATGCTCTCAAAGGGTCACTC | GTCGCATTCTCTGCCTGTTAG | CACTGAAAGTGACCCACACG | ATTAATCACAATGCTCCGGC |
| CG17687 | XR | CTCAAGTCCTGCGACAACTTC | CTCCAGGTGAGCAGATTCTTG | GATCACATTCCGAGTGCTTG | TCGAAGTGGATCATGCAGAG |
| CG18265 | XR | GACATCCCTAAGCCCTTTCAG | TCTTTGTTGCAGATGTTGCAC | AGGAGAAGCTAAACGGGTCC | GCGTACTGGTTCTCCAGCTC |
| CG1915 | XR | GTGCACATGAACAAGCAACAG | ACTGAACACTGGTGGCCTTC | TCAACACCATTCAGAGCACC | GTGTGGATGCTGTCCTCCTC |
| CG32138 | XR | CCAGAAACGGAAAATGGTAGG | GTCAGCTCCTCCTCGAGATTC | GTCGATCATGTGCCTAAGGG | CCCAGGATGATCTCATGACC |
| CG32210 | XR | GCAGAAGAAGTTGTCGTGGAC | CAGAATGGTGGCACTTTCTTC | GAAGATCTAGCCAACGCCAG | ACGCCTGCACTAACAGATCC |
| CG32296 | XR | GCTAATCTGTTCGAGGAGCTG | GGAGGATTGCTTGTTTCCATC | ATCAGTCCATACTGCCCTCG | AGCATCCCCAAAGTTCAGTG |
| CG32311 | XR | GTGATTGTGGGTCAACCAGAG | GTCATCTCCTTGCTCTTGTGC | AAGCTGATCCTGAACAACGC | TGAAGAGCTCCCTGAACTCG |
| CG32333 | XR | GCAGGAGATGCACAAGAAGAC | GAGAAGGTATCGCCCTGATTC | TCCGGTGTACCACATCTACG | GGAGGATTCTGCAGGTTCTG |
| CG33158 | XR | GATCCCAAGTTGCAGATCAAG | CGCTATAACCTTCAGGGTTCC | ATTCGTTTTCGTTGCCTTTG | ACATGTAGAGGTCCCCGATG |
| CG4998 | XR | GCTACGAGGGACCCTACAAAC | CATCCTTCTTGGTCTCTGCTG | GTCAAAGTTCCCAGTGTCCC | TCGGTTTGTAGATGTCACCG |
| CG5589 | XR | GAACTGCAAAAGATCACGGAG | TCCTGCGTGAAGAAGGTAATG | TGCAACAACAACCTCCTCTG | ACAAGGTTCTTGAGATGCCG |
| CG5661 | XR | AGATTCATGTGCTCTTCGTGG | AGACCAGTCCGACTCCGTATC | AGACCGATAGCAACTGCCTG | TCCTGCGACGAATCTTAACC |
| CG5841 | XR | TGGACAGGCAGAATGTAAACC | GGCTATTCAACTGATTGCTGC | TCATGCATCAATCGTCTTCG | GCACTCCTCCAGACTGGTTC |
| CG6445 | XR | GAACTACACTGCTCGAGTGGC | TTCGAAATTTGGATGGTTGTC | ACTGATTGACGGAGCCATTC | TGGTAACGACTGTTCTCCCC |
| CG6451 | XR | AACGATACGATCATCCTGTGC | ACCTTGATGCTGATGCTATCG | AGCTGACGTCCATTAATCCC | TCCCAGTCACATACCAAACG |
| CG6915 | XR | CAGTCCTCCTGTCACAGATCC | GCTCGTGTGTATCTCTAGGCG | GGAAGAGGTCTCCAGTGTGG | GAGATACTGGGCGTCAGCTC |
| CG7020 | XR | AACGGCATGCATGTTATTTTC | ATCCTTCTAAAAATCGACGCC | GCACGGCGTAGTAAGAGTGG | ATTTGTCATTCCATCGAGCC |
| CG7036 | XR | ACACGTCAGAACTCCACCAAC | CAATCGGCTCTAGGACACAAG | ACAGAGATAGAGCCCGCAAC | GTCAATGATGTCCGCCAAC |
| CG7051 | XR | GCGTGTACGAATATTTGACGG | AGAACTCCATGACGCTCACAC | TCAGCGACGTTAGTTTCTGC | CCGTCACTGGTATCGGGTAG |
| CG7177 | XR | GCGACGACTACGAGAAGATTG | GCAATTTGCTGCTGAAGAATC | TCCATGCAGCAAAAGTCAAC | CCATTTGATGTTGTTGTGGC |
| CG7338 | XR | TACATGAATCTTCCGCGTTTC | GTACTTCTGGAATCGCTCGTG | TCTGCACAACGTGGAGAATC | CATTTTCTCCATCGCGAGAC |
| CG7441 | XR | CAGCACTTAGCGAGGACATTC | CTCCTCTCCATACTCCGGTTC | TGCTAGAGATTGGAGCCGAG | CTCCGCTCATTACGGTTCTC |
| CG7479 | XR | GGACATTATCAATTTGCAGCG | CACATCGGCGAGAATAATCAG | TGATCTCACGACAGCGCTAC | CGACAAACGTATCCATGGTG |
| CG7597 | XR | TACACGACACCAAACTCACCC | GTATGTGCCTTCACCGATTTG | TCGTAGGTGTAGGTGCAACG | CCAGGATTATGCACAAGCAG |
| CG7839 | XR | AAGAATGTGATGCAGGGTCTG | ATGGTAGTGCTGACGCAAAAG | CAAAGTCCTCGTCCAGAAGG | AATCGATCCGATTTCTCCG |
| CG7879 | XR | AGAGCAGCGTCGAACAGTC | TTGTTGTTGTTGTGGATGCTG | ACATAGAGTTCTCGCGGGTG | GACCAACGACATCTTGAGGC |
| CG7915 | XR | CTGGAGAAGATCAACGAGGTG | GCGTTTGAGATTTTGCAGTTC | AGGAGGAGATGATCCTGCG | CAGCCAGACTTCAGGACCTC |
| CG8177 | XR | AATCCAATGGAAAAGACCCAC | GCGTTACGGAGCGTACAGTAG | TATTCTCGGCCTTGATCACC | GATTAGTGGGCAGCTTCGAG |
| CG8742 | XR | GCGATACAGAAGAAGCCTGTG | ATAGCTGACACTGTTCCCGTG | AGCGAGTCTAGTACGCCAGC | GTTTTTGGACAGCTTGGAGG |
| CG9007 | XR | ACCATCAGACTGTGGATCAGG | AGTTCCTTTTTCTCACGCTCC | CTCCCCTTGCAGAACAATTC | GGCTGTGTCTGTGTCTGTGG |
| CG9279 | XR | CAGTTCAGCGTTTCATCTTCC | TACTGATGGTCTGGTTGAGGG | GGAAGCTGAGTGGAAGCAAG | TCCTTCAGTTCGTCTGCCTC |
| CG9311 | XR | CAATGAGTCCCTCAGCTTCAC | GTACACATCGTAGGAGGCTGC | TGAACATCAACGAGGAGCAG | ATGTGGGCATTGGGTGTG |
| CG9674 | XR | TGTGATTGTGGGCAATGTATG | CGAAGACGGTCACAAAGTGTC | CGAATACCAGAAGGCTCTGC | TCCCGCTTGTACTTGACAAAG |

Supplementary Table 2. Summary statistics of X-linked loci in *D. miranda* and divergence to *D. pseudoobscura*.

|  |  |  | Synonymous Sites |  |  |  |  |  | Replacement Sites |  |  |
| --- | --- | --- | --- | --- | --- | --- | --- | --- | --- | --- | --- |
| Locus | No. Sites | S | π (%) | D_xy_ (JC) pse | Taj D |  | No. Sites | S | π (%) | D_xy_ (JC) pse | Taj D |
| CG8177 | 296.8 | 14 | 1.57 | 3.52 | 0.19 |  | 852.2 | 2 | 0.03 | 0.13 | -1.48 |
| CG7915 | 274.7 | 2 | 0.10 | 1.15 | -1.48 |  | 868.3 | 0 | 0.00 | 0.00 | nan |
| CG7839 | 268.1 | 8 | 0.51 | 4.90 | -1.73 |  | 814.9 | 4 | 0.10 | 0.42 | -1.16 |
| CG7092 | 264.0 | 7 | 0.74 | 6.67 | -0.41 |  | 846.0 | 1 | 0.02 | 0.13 | -1.16 |
| CG7020 | 279.2 | 5 | 0.60 | 2.21 | 0.19 |  | 839.8 | 0 | 0.00 | 0.00 | nan |
| CG6775 | 170.2 | 3 | 0.45 | 5.12 | -0.57 |  | 564.8 | 7 | 0.28 | 1.96 | -1.02 |
| CG4857 | 258.7 | 0 | 0.00 | 2.76 | nan |  | 821.3 | 2 | 0.05 | 0.39 | -0.96 |
| CG4557 | 257.7 | 0 | 0.00 | 3.98 | nan |  | 834.3 | 3 | 0.07 | 0.27 | -1.28 |
| CG32333 | 246.1 | 3 | 0.47 | 5.98 | 0.65 |  | 746.9 | 4 | 0.09 | 0.45 | -1.48 |
| CG32311 | 262.6 | 18 | 2.43 | 5.03 | 0.45 |  | 865.4 | 2 | 0.05 | 0.02 | -0.96 |
| CG2175 | 243.3 | 8 | 0.74 | 5.86 | -1.10 |  | 824.7 | 7 | 0.17 | 0.49 | -1.37 |
| CG2025 | 262.8 | 10 | 1.21 | 6.17 | 0.03 |  | 886.2 | 1 | 0.02 | 0.57 | -1.16 |
| CG1915 | 266.7 | 16 | 2.14 | 4.10 | 0.48 |  | 882.3 | 1 | 0.02 | 0.01 | -1.16 |
| CG18265 | 256.5 | 8 | 0.63 | 3.07 | -1.37 |  | 856.5 | 5 | 0.14 | 0.56 | -0.78 |
| CG17758 | 271.7 | 5 | 0.55 | 6.50 | -0.20 |  | 814.3 | 0 | 0.00 | 0.12 | nan |
| CG17687 | 259.3 | 7 | 0.66 | 4.10 | -0.83 |  | 871.7 | 2 | 0.03 | 0.94 | -1.48 |
| CG17286 | 272.2 | 3 | 0.16 | 3.46 | -1.67 |  | 813.8 | 0 | 0.00 | 0.12 | nan |
| CG8465 | 270.5 | 3 | 0.20 | 3.13 | -1.28 |  | 884.5 | 1 | 0.02 | 3.24 | -1.16 |
| CG1554 | 238.8 | 3 | 0.44 | 2.90 | 0.33 |  | 787.2 | 0 | 0.00 | 0.00 | nan |
| CG1517 | 268.0 | 15 | 1.51 | 3.24 | -0.61 |  | 851.0 | 0 | 0.00 | 0.00 | nan |
| CG15804 | 244.1 | 3 | 0.28 | 2.23 | -0.89 |  | 850.9 | 0 | 0.00 | 0.35 | nan |
| CG14967 | 250.1 | 5 | 0.44 | 3.57 | -1.04 |  | 856.9 | 0 | 0.00 | 0.23 | nan |
| CG1634 | 257.5 | 1 | 0.06 | 1.60 | -1.16 |  | 825.5 | 0 | 0.00 | 0.00 | nan |
| CG14060 | 260.4 | 2 | 0.16 | 5.25 | -0.96 |  | 861.6 | 3 | 0.06 | 2.84 | -1.28 |
| CG13917 | 155.8 | 6 | 1.20 | 5.37 | -0.06 |  | 504.2 | 0 | 0.00 | 0.00 | nan |
| CG13908 | 261.1 | 9 | 1.11 | 2.25 | 0.05 |  | 827.9 | 3 | 0.08 | 0.41 | -0.96 |
| CG17150 | 212.5 | 7 | 0.53 | 6.09 | -1.81 |  | 744.5 | 0 | 0.00 | 1.08 | nan |
| CG13900 | 266.1 | 3 | 0.34 | 6.91 | -0.14 |  | 852.9 | 0 | 0.00 | 0.35 | nan |
| CG1372 | 248.9 | 6 | 0.91 | 3.59 | 0.68 |  | 816.1 | 6 | 0.18 | 0.35 | -0.87 |
| CG13366 | 265.9 | 3 | 0.16 | 3.55 | -1.67 |  | 862.1 | 0 | 0.00 | 0.12 | nan |
| CG12983 | 253.7 | 5 | 0.65 | 2.87 | 0.17 |  | 886.3 | 4 | 0.13 | 2.34 | -0.24 |
| CG12982 | 248.3 | 6 | 0.73 | 2.61 | -0.14 |  | 813.7 | 9 | 0.27 | 3.32 | -0.89 |
| CG9774 | 254.4 | 3 | 0.17 | 4.13 | -1.67 |  | 861.6 | 0 | 0.00 | 0.23 | nan |
| CG9674 | 237.6 | 1 | 0.06 | 4.36 | -1.16 |  | 764.4 | 1 | 0.03 | 0.02 | -0.34 |
| CG11759 | 246.2 | 1 | 0.11 | 2.96 | -0.34 |  | 794.8 | 1 | 0.02 | 0.13 | -1.16 |
| CG9311 | 253.8 | 6 | 1.13 | 2.69 | 1.80 |  | 835.2 | 1 | 0.05 | 0.33 | 0.84 |
| CG9279 | 248.8 | 12 | 1.05 | 2.45 | -1.25 |  | 834.2 | 1 | 0.02 | 0.01 | -1.16 |
| CG9198 | 281.7 | 5 | 0.60 | 4.82 | 0.26 |  | 858.3 | 0 | 0.00 | 0.12 | nan |
| CG9059 | 15.3 | 1 | 0.94 | 14.88 | -1.16 |  | 44.7 | 0 | 0.00 | 0.00 | nan |
| CG11122 | 267.5 | 5 | 0.55 | 3.75 | -0.24 |  | 824.5 | 3 | 0.08 | 0.37 | -0.89 |
| CG9007 | 248.8 | 6 | 0.43 | 3.53 | -1.54 |  | 846.2 | 0 | 0.00 | 0.83 | nan |
| CG8742 | 259.6 | 11 | 1.43 | 3.09 | 0.24 |  | 817.4 | 0 | 0.00 | 0.00 | nan |
| CG11409 | 265.0 | 4 | 0.46 | 3.00 | -0.10 |  | 803.0 | 3 | 0.13 | 0.33 | 0.26 |
| CG8590 | 256.5 | 6 | 0.43 | 5.49 | -1.50 |  | 904.5 | 0 | 0.00 | 1.34 | nan |
| CG8128 | 279.5 | 0 | 0.00 | 4.42 | nan |  | 887.5 | 4 | 0.10 | 1.65 | -1.02 |
| CG11250 | 295.7 | 2 | 0.18 | 2.66 | -0.44 |  | 865.3 | 1 | 0.02 | 0.12 | -1.16 |
| CG8062 | 260.8 | 10 | 0.94 | 4.20 | -0.89 |  | 807.2 | 0 | 0.00 | 0.37 | nan |
| CG7879 | 239.3 | 3 | 0.34 | 4.55 | -0.42 |  | 780.7 | 0 | 0.00 | 0.77 | nan |
| CG7597 | 223.1 | 12 | 1.75 | 4.45 | 0.09 |  | 718.9 | 4 | 0.12 | 0.21 | -1.02 |
| CG10129 | 270.5 | 12 | 1.28 | 5.82 | -0.36 |  | 899.5 | 4 | 0.06 | 0.57 | -1.80 |
| CG10236 | 262.2 | 8 | 0.90 | 2.24 | -0.26 |  | 826.8 | 0 | 0.00 | 0.61 | nan |
| CG7479 | 242.5 | 2 | 0.22 | 2.21 | -0.44 |  | 747.5 | 2 | 0.04 | 0.56 | -1.48 |
| CG7441 | 242.8 | 1 | 0.06 | 2.97 | -1.16 |  | 798.2 | 2 | 0.08 | 1.32 | 0.04 |
| CG7338 | 250.5 | 4 | 0.52 | 5.78 | 0.13 |  | 877.5 | 0 | 0.00 | 0.34 | nan |
| CG7177 | 241.2 | 2 | 0.22 | 3.27 | -0.44 |  | 922.8 | 2 | 0.03 | 0.45 | -1.48 |
| CG7051 | 283.7 | 10 | 0.87 | 5.19 | -0.86 |  | 889.3 | 0 | 0.00 | 0.90 | nan |
| CG7036 | 237.6 | 3 | 0.32 | 3.57 | -0.57 |  | 752.4 | 0 | 0.00 | 0.27 | nan |
| CG6915 | 272.0 | 11 | 1.46 | 5.33 | 0.54 |  | 802.0 | 0 | 0.00 | 0.12 | nan |
| CG6451 | 235.4 | 0 | 0.00 | 6.66 | nan |  | 760.6 | 1 | 0.02 | 0.01 | -1.16 |
| CG6445 | 290.5 | 9 | 0.73 | 5.93 | -0.98 |  | 870.5 | 2 | 0.05 | 0.14 | -0.96 |
| CG12149 | 268.8 | 19 | 2.06 | 3.76 | -0.37 |  | 793.2 | 3 | 0.05 | 0.03 | -1.67 |
| CG5841 | 269.6 | 5 | 0.50 | 3.33 | -0.49 |  | 837.4 | 1 | 0.02 | 0.49 | -1.16 |
| CG5661 | 137.5 | 10 | 2.34 | 3.29 | 0.03 |  | 462.5 | 0 | 0.00 | 0.22 | nan |
| CG12467 | 264.4 | 0 | 0.00 | 5.49 | nan |  | 788.6 | 0 | 0.00 | 0.25 | nan |
| CG5589 | 278.7 | 4 | 0.53 | 2.55 | 0.56 |  | 864.3 | 1 | 0.06 | 0.05 | 1.43 |
| CG4998 | 275.6 | 4 | 0.38 | 3.77 | -0.59 |  | 807.4 | 3 | 0.16 | 0.51 | 1.25 |
| CG3806 | 258.0 | 3 | 0.25 | 5.78 | -0.96 |  | 840.0 | 0 | 0.00 | 0.48 | nan |
| CG3620 | 236.2 | 3 | 0.18 | 1.81 | -1.67 |  | 810.8 | 0 | 0.00 | 0.00 | nan |
| CG33158 | 274.0 | 5 | 0.56 | 4.10 | -0.10 |  | 866.0 | 7 | 0.19 | 0.80 | -0.87 |
| CG32704 | 267.7 | 0 | 0.00 | 4.62 | nan |  | 821.3 | 1 | 0.02 | 0.48 | -1.16 |
| CG32562 | 273.4 | 2 | 0.19 | 4.08 | -0.53 |  | 872.6 | 0 | 0.00 | 0.69 | nan |
| CG32560 | 260.7 | 16 | 2.25 | 4.60 | 0.60 |  | 786.3 | 0 | 0.00 | 0.00 | nan |
| CG32527 | 268.4 | 1 | 0.05 | 5.43 | -1.16 |  | 835.6 | 2 | 0.03 | 0.98 | -1.48 |
| CG12737 | 269.0 | 9 | 0.99 | 4.37 | -0.26 |  | 799.0 | 1 | 0.03 | 0.99 | -0.34 |
| CG32210 | 248.9 | 3 | 0.60 | 5.26 | 1.75 |  | 846.1 | 2 | 0.08 | 1.16 | 0.04 |
| CG3193 | 252.0 | 0 | 0.00 | 5.34 | nan |  | 837.0 | 1 | 0.03 | 0.50 | -0.34 |
| CG17149 | 275.0 | 4 | 0.42 | 5.27 | -0.30 |  | 829.0 | 0 | 0.00 | 0.36 | nan |
| CG10107 | 292.4 | 1 | 0.05 | 4.56 | -1.16 |  | 805.6 | 0 | 0.00 | 1.00 | nan |
| CG32138 | 236.1 | 4 | 0.62 | 3.21 | 0.50 |  | 795.9 | 1 | 0.02 | 0.26 | -1.16 |
| CG3003 | 272.0 | 4 | 0.36 | 2.84 | -0.70 |  | 775.0 | 7 | 0.18 | 0.76 | -1.37 |
| CG32296 | 291.6 | 4 | 0.42 | 4.52 | -0.10 |  | 818.4 | 0 | 0.00 | 0.12 | nan |
| CG10990 | 268.1 | 0 | 0.00 | 3.83 | nan |  | 832.9 | 2 | 0.12 | 1.67 | 1.51 |
| CG2841 | 251.8 | 3 | 0.35 | 5.14 | -0.24 |  | 750.2 | 2 | 0.04 | 1.77 | -1.48 |
| CG3093 | 268.7 | 1 | 0.16 | 2.16 | 0.84 |  | 850.3 | 1 | 0.02 | 0.48 | -1.16 |
| CG12734 | 250.9 | 2 | 0.32 | 4.79 | 0.75 |  | 874.1 | 0 | 0.00 | 0.11 | nan |
| CG2194 | 272.6 | 16 | 1.75 | 5.49 | -0.25 |  | 831.4 | 2 | 0.08 | 0.17 | 0.04 |
| CG9900 | 254.1 | 2 | 0.11 | 3.27 | -1.48 |  | 828.9 | 2 | 0.06 | 1.13 | -0.44 |
| CG12199 | 241.2 | 13 | 1.84 | 4.33 | 0.28 |  | 718.8 | 0 | 0.00 | 0.00 | nan |
| CG3078 | 284.3 | 0 | 0.00 | 3.60 | nan |  | 798.7 | 2 | 0.09 | 0.70 | 0.23 |
| CG12132 | 272.4 | 4 | 0.25 | 4.29 | -1.48 |  | 807.6 | 0 | 0.00 | 0.50 | nan |
| CG2984 | 233.5 | 3 | 0.24 | 4.09 | -1.28 |  | 774.5 | 4 | 0.12 | 1.76 | -0.85 |
